# Carcass Characteristics, Meat Quality and Nutritional Composition of Kadaknath, a Native Chicken Breed of India

**DOI:** 10.3390/foods11223603

**Published:** 2022-11-11

**Authors:** Santosh Haunshi, Suresh Devatkal, Lawrence Leslie Leo Prince, Rajkumar Ullengala, Kannaki Ramasamy, Rudranath Chatterjee

**Affiliations:** 1 ICAR-Directorate of Poultry Research, Rajendranagar, Hyderabad 500030, India; 2 ICAR-National Research Centre on Meat, Chengicherla, Hyderabad 500092, India

**Keywords:** native chicken, Kadaknath, broiler, carcass, meat quality, nutrition, amino acids

## Abstract

The study was carried out to investigate the carcass and meat quality traits and nutritional profile of the meat of the Kadaknath, a unique native chicken breed in comparison with commercial broilers. The yield of the carcass, breast and giblets of the Kadaknath was lesser (*p* < 0.01), while that of the legs, wings, back, and neck was higher (*p* < 0.01) than broilers. The meat of the Kadaknath was significantly (*p* < 0.0001) darker (42.44, 50.92) and more yellow (6.23, 8.99) than broilers. The decline in pH of the meat was lower (*p* < 0.001) in the Kadaknath compared to broilers. Kadaknath meat had more protein and less fat, moisture and ash content than broilers (*p* < 0.01). Furthermore, it was richer (*p* < 0.01) in 11 amino acids, including those which are known to impart a sweet and umami taste, than the meat of broilers (3 amino acids). Both genotypes were almost similar in meeting the daily requirements of indispensable amino acids of adult human. The study concluded that the Kadaknath differed in carcass and meat quality characteristics from the broilers, and the nutritional quality of Kadaknath meat in terms of high protein and less fat and higher content of amino acids (tasty type) was better in Kadaknath meat as compared to broiler meat.

## 1. Introduction

The meat from slow-growing native chicken breeds is considered an alternative to commercial white broiler meat. In recent years, the consumer demand for the meat of native chickens has been growing immensely. This rising demand is mostly attributed to better taste and flavour, and low-fat content. The meat of the slow-growing native chickens is considered healthy due to their slow growth and environmentally friendly as they do not require feeding of antimicrobial/antibiotic growth promoters, that is a usual practice in the rapidly growing broilers. Furthermore, native chickens are hardy and reported to be resistant to most of the commonly occurring bacterial and parasitic diseases [1,2] and therefore they are least likely to be administered with antimicrobial agents for prophylactic and therapeutic purposes.

Kadaknath is the unique and most popular breed among the notified and registered chicken breeds of India. These birds can thrive under low input backyard and free-range farming in tropical conditions that are otherwise harsh to the improved exotic breeds. Kadaknath is a slow-growing bird, and its growth rate is lesser than that reported for Aseel, a famous native chicken breed of India [3]. The meat of this breed is considered to be a delicacy due to its perceived taste and flavour. Furthermore, the meat of this breed is presumably known to have better nutrient profile as compared to commercial broilers. Therefore, Kadaknath meat is being sold in niche market with a premium price (2 to 3 times) than that of broiler meat. This breed has attracted tremendous attention due to its unique black colour hyper-pigmentation. The hyper-pigmentation is due to the *fibromelanosis* (*Fm*) phenomenon caused by the excessive deposition of eumelanin in almost all parts of the body including muscles and internal organs. The meat of this breed is also black [4]. The black pigmentation character may be similar to the one noticed in the other two famous breeds; Silkie breed from China and Ayam Cemani of Indonesia [5]. Kadaknath is a slow growing native chicken with low to moderate egg production potential [6].

Meat quality is a function of the genotype, nutrition and rearing practises. Previous studies have reported the differences in carcass and meat quality traits between slow-growing and fast-growing chicken lines [7,8]. Further, the genotype of birds (growth rate) is also known to play an important role in the nutrient composition (proximate composition and amino acid profile) of the meat. Previously, it was reported that the meat of slow growing birds (Thai indigenous chickens and meat type birds) had more protein, less fat and ash content as compared to the meat of fast growing commercial broilers and they differed in the amino acid composition as well [9,10]. It was also reported that the fat and cholesterol content of slow growing Thai native chicken’s meat was lower as compared to the fast-growing Rhode Island Red chickens [11]. The differences were also observed in the colour of muscles between slow growing native chicken (Aseel) and broilers [12,13]. Recently, it was reported that the genotype of birds not only affected the proximate principles but also the amino acid composition of meat [14]. The meat of Polverara chickens was reported to contain more protein and less fat and had a better profile of indispensable amino acids as compared to commercial broilers [14].

The meat of Kadaknath is reported to be a rich source of functional biomolecules such as carnosine [15]. However, there is a little information about the nutritional value of the Kadaknath meat *vis à vis* broiler chicken. It is hypothesized that the meat of this slow-growing Kadaknath differs from that of fast-growing chickens for various carcass and meat quality traits including the nutritional profile. Therefore, the aim of the present study was to characterize the Kadaknath breed for carcass and meat quality traits, including nutritional and amino acid profile in comparison with the broilers.

## 2. Materials and Methods

The study was carried out at the experimental unit of the poultry research institute of ICAR, Hyderabad (17°20′ N, 78°30′ E), India. The Institute Animals Ethics Committee (IAEC) had approved the complete experiment. A total of 360 Kadaknath chicks were hatched and raised on the floor in open-sided house. Chicks were vaccinated against commonly occurring viral diseases (M.D., N.C.D., IBD, fowl pox and I.B.). During the first 8 weeks of age, chicks were given starter ration, and during the growing period (9–20 weeks), grower ration was given to grower birds in sufficient quantity. From 20 weeks onwards, male Kadaknath birds were reared in male cages, and breeder ration was given in ad lib quantity. The detailed information about the feed formulations used for feeding Kadaknath chickens is given in Table 1.

### 2.1. Age and Type of Birds at Slaughter

At the age of 27 weeks, 20 male birds of Kadaknath were randomly selected and fasted for 12 h. A total of 12 live broilers (male) of about 5 weeks of age were purchased from the retail market. The Suguna strain of broiler was used for the comparative study. The maize, soybean meal-based starter ration (3000 kcal/kg energy (ME) and 22.5% protein (CP)) during the starting period and finisher ration (3250 kcal/kg ME and 20% CP) during the finishing period are normally used for feeding the broilers by the farmers. The Kadaknath chickens and commercial broilers were selected on an equal body weight basis in such a way that their average body weights (1700 and 1760 g) were not significantly different from each other for the comparative study (Table 2). Upon arrival at the slaughterhouse, birds were given adequate rest and weighed individually before slaughter.

### 2.2. Slaughtering and Dressing

Birds were slaughtered by the exsanguination method through manual cutting of the carotid arteries. Subsequently, scalding was carried out at 54 °C for 4 min and de-feathering was carried out by keeping the skin intact. The inspection of the meat was performed after the evisceration process. The weight of each dressed carcass, heart, gizzard, liver, testes and other non-edible parts (head, shank/feet, feathers and blood and abdominal fat) was noted. After overnight chilling (6.0 ± 1.0 °C), the dressed carcass was cut into main parts and the weight of each part was documented. The yield of the carcass, organs and all the cut-up parts including non-edible parts was expressed as the proportion of live bodyweight.

### 2.3. Meat Quality Traits

The pH of the breast muscles was recorded at 45 min, 24 h and 48 h post mortem by standard procedure. The pH of the meat was measured using a probe pH meter (Hanna Instruments, Inc., Woonsocket, RI, USA, 2019 make) having a glass electrode attached to a stainless-steel sharp probe to insert deep into the muscle tissue. The instrument was calibrated with a standard pH of 4 and 7 before taking measurements. The pH readings of the probe method were confirmed with the laboratory method. Briefly, muscle samples were grounded and then homogenized with distilled water in 1:5 (wt./vol.) ratio. The pH of this homogenate was recorded by dipping the glass electrode of the pH-meter (Eutech, Maharashtra, India, PH Tutor, Cyberscan, 2017 make). The colour of the breast and thigh meat on both (inner and outer) sides was determined at 2 places each (total of four locations for each breast and thigh sample) using a colour reader (Konica Minolta, CR-10 Plus, Tokyo, Japan). The colorimeter was calibrated throughout the study using standard white and black tiles. The colour readings were recorded as per the colour profile of International Commission on Illumination (CIE) system after exposing the meat samples to ambient air for 20 min. The parameters of L* (lightness), a* (redness), and b* (yellowness), C* (chroma = √(a*^2^) + (b*^2^)) and H* [hue = tan^−1^ (b*/a*)] were recorded as per the standard methods [16]. Overall colour difference (∆E) was calculated using the formula, ∆E = [(∆L)^2^ + (∆a)^2^ + (∆b)^2^]1/2; the values of ∆L, ∆a, and ∆b are the difference in colours (light, red and yellow) between respective muscles of Kadaknath and broiler, or between thigh and breast muscles within the breed. Meat and bones of breast, thigh and drumstick were separated and weighed individually. Meat–bone ratio was calculated after the separation of muscles from bones manually and by weighing bones and muscle separately.

### 2.4. Proximate Principles

The proximate principles such as moisture, protein, fat and ash of breast muscles were determined using standard AOAC and BIS procedures. The level of moisture in the meat was assessed using the oven method (930.15: AOAC). Crude protein was estimated by the Kjeldahl distillation procedure (984.13: AOAC). The Soxhlet apparatus—solvent extraction method (920.39: AOAC) was used to estimate the fat content [17] while ash was determined with a muffle furnace (IS 14827, Bureau of Indian Standards: BIS). Analysis of proximate principles was carried out at AFAQAL facility of VC & RI, Namakkal, India.

### 2.5. Estimation of Amino Acids

The amino acid profile of breast meats of two genotypes was determined in duplicate by the UPLC-MS (ultra-high-performance liquid chromatography-mass spectrometry) procedure [18]. In brief, 100 mg of dried meat samples was taken in a Thumburgs tube (Vensil, Thumburg tube 20 mL Joint 19/26 Cat. No. 1995) and 1.5 mL of 6M hydrochloric acid was added to it, mixed thoroughly and heated under vacuum at 110 °C for 6 h in a hot air oven. At room temperature, the volume of the samples was made to 10 mL using distilled water. The content was centrifuged (REMI cooling centrifuge C-24 BL) at 10,000× *g* for the duration of 15 min. From this 0.1 mL of supernatant was collected, dried completely in rotary flask evaporator (Heidolph laborota 4000, at 90 rpm speed and temperature of 45 °C), and dissolved in 0.1% formic acid in 20% methanol. Subsequently, the dissolved supernatant was filtered through the 0.22 μm nylon filter membrane (product Code- PV0213SF-100PB Randisc PVDF SF from Rankem) and 5 μL of the sample was injected onto the column (Waters Acquity UPLC BEH C18 Column, 1.7 μm particle size, 2.1 × 50 mm column) of UPLC-MS (Waters UPLC H-class system) with column oven temperature maintained at 25 °C. The elution was monitored using TQD-MS/MS system (Waters, Milford, MA, USA) which was optimized for analysing amino acids profile. For extraction of free amino acids, 0.1 g of the sample was homogenized with 20 mL of 0.1% formic acid in 20% methanol. Subsequently, the homogenized sample was centrifuged at 10,000× *g* for 15 min, supernatant was collected, and the volume was made to 10 mL with 0.1% formic acid in 20% methanol. The supernatant was filtered through 0.22 μm nylon filter membrane and injected onto UPLC-MS/MS system to estimate the tryptophan, the unbound or free amino acid. The amino acid analysis of meat samples was carried out at the sophisticated analytical instrument facility, ICAR-IIHR, Bengaluru.

### 2.6. Statistical Analysis

The descriptive statistics of various traits were determined by following established methods. Student’s ‘t’ test was used to determine the significant difference in the means of various traits between tow genotypes or sex. Pearson’s correlation coefficients (two-tailed) were calculated to study the correlation amongst different variables of meat quality and proximate composition.

## 3. Results

### 3.1. Carcass Characteristics

Live body weight of both Kadaknath and broilers was not significantly different. However, carcass weight was significantly higher in broilers (Table 2). In cut-up parts, the weight of the breast was significantly (*p* < 0.0001) higher in broilers while weights of the legs, back and neck were significantly (*p* < 0.05) higher in the Kadaknath breed. Therefore, the yield of carcass (dressing percentage) and breast was significantly (*p* < 0.01) higher in broilers. Absolute weights of organs such as liver, heart and gizzard (giblets) and their yield were significantly (*p* < 0.001) lesser in the Kadaknath breed when compared to those of broilers at 27th weeks of age.

### 3.2. Meat Quality Traits

The findings of the meat quality study (Table 3) revealed that there was no significant difference in initial pH (45 min) of muscles between Kadaknath and broilers. However, pH of muscles recorded at 24 and 48 h post-slaughter was significantly (*p* < 0.01) higher in Kadaknath than that of broilers. The decline in pH of meat both at 24 and 48 h post-slaughter was highest (*p* < 0.01) in broilers as compared to the Kadaknath. The weight of the meat from breast part was significantly (*p* < 0.0001) higher in broilers whereas it was significantly higher (*p* < 0.0001) in the drumstick part of Kadaknath. When breast, thigh and legs taken together, the higher (*p* < 0.0001) quantity of meat was recorded in broilers as compared to the Kadaknath. Thigh bones of Kadaknath were significantly (*p* < 0.02) heavier than broilers. The meat–bone ratio of breast and thigh parts was significantly (*p* < 0.001) higher in broilers while that of the drum was significantly (*p* < 0.001) higher in Kadaknath. Overall, a higher meat–bone ratio was seen in broilers as compared to the Kadaknath.

**Figure 1:**
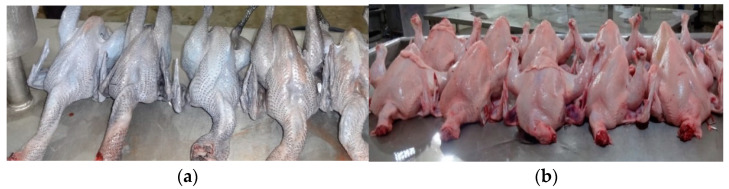
Dressed carcasses of Kadaknath (**a**) and broiler (**b**) chickens.

Means and standard errors of colour parameters of breast and thigh muscles of Kadaknath and broilers are given in Table 4. The breast and thigh muscles of Kadaknath were significantly (*p* < 0.0001) darker and less yellow than muscles of respective parts of broilers. The colour intensity and chroma (saturation index) of breast and thigh muscles were significantly (*p* < 0.0001) higher in broilers than in respective muscles of Kadaknath (Figure 1 and Figure S1). However, there was no difference in redness (a*) and hue of meat of Kadaknath and broilers. Within a genotype, the breast muscles of Kadaknath were significantly (*p* < 0.0001) lighter, less red, more yellow and had more chroma than its thigh muscles. Similarly, in broilers, the breast muscles were significantly (*p* < 0.05) lighter, more yellow and had less hue than the thigh muscles. The average total colour difference (ΔE) between the thigh muscles of Kadaknath and broilers was 13.19 ± 1.18 and that of the breast muscles of Kadaknath and broilers was 8.94 ± 1.91. The ΔE value between breast and thigh muscles of Kadaknath was 8.07 ± 0.64 and that of breast and thigh muscles of broiler was 6.14 ± 0.71. All four ΔE were higher than 4 indicating that the colour difference between respective muscles (thigh or breast) of broilers and Kadaknath and between thigh and breast muscles within the breed (Kadaknath and broilers) can be visually appreciated. The total colour difference was particularly high for the thigh muscles of Kadaknath and broilers as compared to the breast muscles of these two genotypes.

### 3.3. Nutrient Composition Analysis

The nutrient composition of breast meat of Kadaknath and broilers are reported in Table 5. The breast meat of Kadaknath had significantly more protein (*p* < 0.0002) content and less moisture (*p* < 0.0001), less fat (*p* < 0.007), less ash (*p* < 0.031) and less gross energy (*p* < 0.0001) content than those of breast meat of broilers.

### 3.4. Relationship between Meat Quality and Proximate Principles

The correlation between moisture and MBR (−0.55) and moisture and ash (−0.52) in the Kadaknath and correlation between pH45m and MBR (−0.59) in broilers was significant (*p* < 0.05). However, there was no signification difference in the trend of association between rest of the meat quality and proximate composition traits among two genotypes. Therefore, the data of both genotypes were pooled and analysed to obtain better statistical power in detecting the relationship among various meat quality and nutrient composition traits (Table 6). Significant (*p* < 0.01) correlations were observed among various meat quality and nutrient composition traits. The pH of meat recorded at 45 min post slaughter was positively correlated with the pH recorded at 24 (pH24h) and 48 h (pH48h) post slaughter. Similarly, the pH24h was positively correlated with the pH48h. pH24h and pH48h were negatively correlated with the moisture content while positively correlated with protein content. pH48h was also negatively correlated with meat–bone ratio (MBR), lightness, yellowness and chroma. MBR was positively correlated with lightness of the meat, fat and ash contents. The lightness of the muscle was positively correlated with yellowness, chroma, moisture, and ash contents. However, the lightness and protein content were negatively correlated. Redness and hue were positively correlated with each other. Yellowness was correlated positively with chroma and moisture content while it was negatively correlated with protein content. Chroma was correlated positively with moisture while being correlated negatively with protein content. Moisture and protein contents of breast meat were negatively correlated with each other.

### 3.5. Amino Acid Profile

The analysis of the composition of amino acids in meat samples revealed that (Table 7) lysine, methionine, phenylalanine, valine and arginine were the most abundant indispensable amino acids in both genotypes. Among dispensable amino acids, glutamic acid, proline and tyrosine were the most abundant in both types of chickens. The Kadaknath meat was rich in leucine and tryptophan while broiler meat was rich in histidine, methionine and threonine indispensable amino acids. It was observed that the Kadaknath meat had significantly higher content of most of the dietary non- indispensable amino acids (except tyrosine) as compared to the broiler meat. Overall, the Kadaknath meat had contained significantly (*p* < 0.001) higher quantity of 11 amino acids in comparison to 3 amino acids in broilers while no significant difference was seen in the content of 5 amino acids. The Kadaknath meat had significantly higher content of amino acids (alanine, asparagine, glutamic acid, glycine and serine) which are known to impart a sweet and umami (savoury) taste to the meat as compared to the broiler meat.

## 4. Discussion

The information about the meat quality traits and nutrient composition of Kadaknath meat *vis à vis* broiler helps the consumers to make an informed decision in purchasing and consuming the meat of this breed. The growth of the Kadaknath like any other native chicken is slow. They attain the market weight of about 1.5 to 2.0 kg (live bodyweight) only by 24–28 weeks of age as compared to 5 to 6 weeks required for broilers to reach this weight. In this study, it was seen that even with similar bodyweight as that of broilers, Kadaknath had significantly lesser dressed weight and hence significantly lesser dressing and breast yield. The lesser yield observed in the Kadaknath breed might be due to less muscle mass and higher percentage of non-edible portions such as head, shank, and blood and feathers as a proportion of live bodyweight of this breed. Further, high muscle and bone mass of broilers might have contributed to the higher dressing yield in broilers. The dressing percentage of Kadaknath was similar to that of other native chickens such as Aseel Peela (69.54, 70.8%) [4,12], and Ghagus (70.2%) [19]. However, the dressing percentage recorded in this study is higher than those observed in other studies for Kadaknath (65.2%) birds slaughtered at a younger age of 20 weeks [20] and Nicobari (65.2%) breed (Haunshi et al., Unpublished).

Breast yield was higher in broilers while the yield of legs, wings, back and neck were higher in Kadaknath. This difference was due to the selection of broilers for higher breast yield. As native birds are required to run faster and fly to escape from predators in the free-range rearing systems, they are naturally selected for stronger legs, wings, neck and back muscles leading to higher proportion in legs, wings, and back (Figure 1). Weights of leg and breast parts of Kadaknath were comparable to those of other native chickens such as Aseel (22.7 and 17.1%), Ghagus (23.4 and 16.3%) and Nicobari (20.51 and 15.91%) breeds [12,20], (Haunshi et al., unpublished). Higher yield of liver, heart and gizzard (giblets) observed in broilers is due to higher feed intake, assimilation of nutrients and metabolism that are required for rapid growth in such a short period of time (35–42 days). Native birds are slow in growth and their feed intake is also very low hence have a lesser percentage of internal organs related to digestion and metabolism as compared to the fast-growing broilers. The giblet percentage observed for broilers in the present study was comparable with those reported for white broilers [13].

The abdominal fat of Kadaknath was also negligible while broilers had more abdominal and subcutaneous fat (Figure S1). Like any other native chickens, Kadaknath birds had less abdominal fat as observed in slow-growing native breeds such as Aseel Peela (0.02%) chicken [4], but it was lower than that reported for Aseel (0.75%) [12] and Nicobari (1.5%) breed. The abdominal fat observed in broilers is similar to the one (1.85%) reported previously for broilers [12]. Significantly higher meat–bone ratio observed in broilers can be explained by the fact that broilers were selected for a high growth rate and hence resulted in higher meat yield. Drum part of Kadaknath has significantly more meat and hence had higher MBR as compared to that of broiler. This finding is desirable and economically significant as Indian consumers prefer the drum part of the chicken leg as compared to the breast meat of the chicken.

Although the weight of the dressed weight and cut up parts was higher in male birds there was no difference in most of the carcass traits including dressing percentage between male and female birds of Kadaknath. These findings were almost similar to the ones observed in Vanashree (evolved from native chicken) chicken [21]. However, the effect of sex on meat quality traits in this breed needs to be investigated.

The colour of the meat plays an important role in consumers’ preference for the meat. Generally, the meat of the Kadaknath is black in colour as compared to any other chickens. Instrumental measurements also showed that both breast and thigh meats of Kadaknath were significantly darker and had more yellowness than broilers. The colour of the chicken meat is influenced by various factors such as muscle type, genotype, age, sex, diet, amount of myoglobin and heme pigments in the muscles, processing, etc. [22]. The meat of the slow-growing chickens is reported to be darker and show more yellow colour as compared to the fast-growing chickens [9,11,12,13]. The lightness, redness and yellowness colour values of breast muscles of fast-growing broilers were reported to be in the range of 53.59 to 55.54, 3.05 to −0.93, 4.97 to 8.48, respectively [7,23,24]. The colour values of breast muscles of broilers observed in our study were almost within this range. The colour values of the Kadaknath breed were comparable to those reported (39.32, 2.49 and 4.02) for Thai indigenous chickens [9], but lesser than that reported for (53.92, 2.30 and 7.66) Aseel breed of chicken [13]. In Kadaknath birds, the excess deposition of melanin pigment in muscles contributed to the dark colour of the meat. Furthermore, the black colour pigmentation appears to reduce the chroma of the Kadaknath meat. To our knowledge, this is the first study to objectively measure the colour of the Kadaknath meat in comparison with the broilers.

The pH of the meat plays a vital role in meat quality. The low ultimate pH may lead to acid meat with undesirable characteristics of pale soft exudate meat as it is negatively correlated with drip loss, cooking loss and texture [25]. The less decline in pH of Kadaknath meat is in a desirable direction and indicates better meat quality. It was reported that pH and colour of muscles are highly correlated. As muscles with lower pH tend to be lighter in colour while muscles with higher pH tend to be darker in colour [7]. Like the present study, Debut [8] observed significant negative correlation of pH24h with lightness and yellowness. Further, Le Bihan-Duval [25] reported negative correlation (genetic) between pH with lightness and yellowness colours of muscles. Colour variables (L*, a* and b*) are related to each other. As observed in the present study, other studies also reported a positive correlation between lightness with yellowness and chroma while a negative correlation between lightness and redness in breast meat of male broilers [8,26]. It was well documented that breast and thigh muscles differ in colour characteristics as breast muscles tend to be lighter, yellower and less red as compared to the leg meats as seen in the Kadaknath breed. In this study, it was observed that breast meat of both Kadaknath and broilers was lighter and less red as compared to their respective thigh meats. Chen et al. [27] observed that irrespective of breeds, the breast meat was lighter and less red as compared to their respective thigh meats.

The Kadaknath meat had a favourable nutrient composition as it had relatively high protein content, less fat, less moisture and less ash content compared to the meat of broilers. The significant difference observed between these two types of birds in the nutrient composition may be attributed to the genetic makeup (breed) and the age difference the birds. It was reported that as the age of the bird increases, the composition of body tissues changes and that the protein content increases and moisture content decrease [28]. There was a considerable variation in the age of the Kadaknath and broilers because of their different market age. Furthermore, selection for rapid growth in broilers resulted in excess fat deposition in muscles as it was observed for the abdominal fat. The protein content of Kadaknath was higher while that of fat and ash was lesser than those reported for the Aseel breed with more or less similar moisture content [29]. Similar to our findings, Thai native chickens were reported to have higher protein, lesser fat and lesser ash as compared to broilers [9]. Likewise, Fanatico [10] reported that the breast muscles of slow-growing birds had more protein and less fat compared to fast-growing ones. The breast muscles of Polverara, a slow-growing native chicken breed of Italy had higher protein and less fat and moisture content as compared to commercial hybrid birds [14]. It appears that growth rate of birds besides genotype, greatly influences the nutrient composition of the chicken meat.

Considerable difference was seen in the amino acid content of breast muscles of Kadaknath and broilers. Out of 19 amino acids investigated, the Kadaknath was significantly higher in the content of 11 amino acids, while the broiler was significantly higher in 3 amino acids content. Similar to the proximate composition, variation seen in the amino acid composition between two types of bids might be due to either the genetic makeup or the age of the birds. The higher content of methionine and lysine in broilers might be due to the supplementation of synthetic amino acids such as lysine and methionine to support the rapid growth of broiler diets. while the male breeder diet fed to Kadaknath had no additional supplementation of these synthetic amino acids.

When the WHO/FAO/UNA recommendations [30] for the daily requirements of indispensable amino acids for a 60 kg adult were taken into consideration, the consumption 100 g meat of Kadaknath or broilers met more than 100% of the daily requirement of lysine, methionine, phenyl alanine, valine, methionine + cysteine, phenyl alanine + tyrosine, and threonine (97% in Kadaknath) amino acids. However, the daily requirement of leucine (49 vs. 40%), histidine (5 vs. 6%) and tryptophan (2 vs. 1%) amino acids was partially met by Kadaknath and broiler meats. Broiler meat had slight edge over Kadaknath meat in meeting the requirement of threonine amino acid while Kadaknath had edge over broiler for leucine amino acid. However, both breeds met almost same level of requirements of histidine and tryptophan amino acids. Therefore, Kadaknath and broilers did not differ considerably in meeting the daily requirements of indispensable amino acids.

Amino acids which are known to provide sweet and umami (savoury) taste attributes to the meat are alanine, asparagine, glutamic acid, glycine, serine and threonine [31,32]. Kadaknath breast meat was significantly higher in these tasty amino acids (with the exception of threonine) as compared to the broilers. Similarly, the meat of the Thai indigenous chickens was reported to have higher glutamic acid content than broiler muscles [8]. Likewise, the Korean native chickens considered to be superior in flavour had higher content of tasty amino acids than broilers [33]. These findings explain the preference or likeness of people for the meat of native chickens including the Kadaknath breed over that of broilers. There is a perception among the consumers that native chicken meat has better taste and flavour as compared to broiler meat and that difference in amino acid content might be contributing the perception of consumers.

## 5. Conclusions

The carcass and meat quality traits and nutrient composition of Kadaknath birds differed from those of commercial broilers. Although both genotypes differed in the contents of both dispensable and indispensable amino acids, there was no considerable difference in meeting the WHO recommended daily requirements (adult human) of indispensable amino acids. The nutrient composition of Kadaknath meat was favourable to the health-conscious consumers as it had a significantly higher quantity of protein with less fat content. Further, the level of amino acids, particularly those known to impart a sweet and umami (savoury) taste to the meat, were higher in the Kadaknath. These findings are important from the consumers’ viewpoint to market the Kadaknath meat as nutritious, healthy and natural alternative meat for consumers in a niche market. Further investigations on the fatty acid profile and mineral (iron) content of breast and thigh meats and microstructure and proteomics of the muscle fibres of the Kadaknath chicken in comparison with fast-growing broilers are required to understand the complete nutritional profile and molecular properties of muscles of Kadaknath chicken.

## Figures and Tables

**Table 1:** Composition of feeds used for the feeding of Kadaknath during various stages of growth.

Ingredients, kg	Chick Starter Ration	Grower Ration	Breeder Ration
Maize	62.25	56.10	66.00
Soybean meal	25.40	24.09	15.00
De-oiled rice bran	7.83	15.32	14.76
Stone grit	1.58	1.86	2.18
Dicalcium phosphate	1.90	1.66	1.21
NaCl salt	0.45	0.45	0.40
DL-Methionine	0.13	0.11	0.00
L-Lysine HCl	0.04	0.00	0.00
Trace minerals	0.084	0.084	0.10
AB2D3K	0.015	0.015	0.00
B complex vitamin	0.015	0.015	0.10
Choline chloride	0.10	0.10	0.10
Toxin binder	0.10	0.10	0.10
Tylosine	0.05	0.05	0.05
Coccidiostat	0.05	0.05	0.00
**Total**	100	100	100
**Nutrient composition, % (calculated basis)**			
ME, kcal/kg	2817	2713	2822
Crude protein	18.00	18.00	14.50
Lysine	0.95	0.93	0.71
Methionine	0.44	0.45	0.30
Calcium	0.99	1.13	1.00
Available phosphorous	0.46	0.44	0.35

**Table 2:** Carcass characteristics of Kadaknath cocks (27th wk) and commercial broilers (5th wk).

Traits	Genotypes		*p* Value
	Kadaknath	Broilers	
Live weight (g)	1707 ± 15.9	1762 ± 54.7	NS
**Carcass composition (g)**			
Carcass weight	1203 ± 12.7	1277 ± 46.9	0.0071
Dressing percentage	70.5 ± 0.45 ^b^	72.4 ± 0.61 ^a^	0.0164
Breast weight	258.4 ± 3.72 ^b^	387.8 ± 18.20 ^a^	0.0001
Legs weight	374.8 ± 7.47 ^a^	346.2 ± 12.40 ^b^	0.044
Wings weight	142.7 ± 2.42	138.2 ± 4.06	0.308
Back weight	260.9 ± 8.35 ^a^	219.7 ± 9.13 ^b^	0.003
Neck weight	54.3 ± 1.66 ^a^	32.9 ± 3.36 ^b^	0.0001
Breast yield (%)	15.1 ± 0.18 ^b^	21.9 ± 0.47 ^a^	0.0001
Legs yield (%)	21.9 ± 0.33 ^a^	19.6 ± 0.30 ^b^	0.0001
Wings yield (%)	8.4 ± 0.12 ^a^	7.9 ± 0.15 ^b^	0.0133
Back yield (%)	15.3 ± 0.52 ^a^	12.4 ± 0.24 ^b^	0.0003
Neck yield (%)	3.2 ± 0.09 ^a^	1.9 ± 0.16 ^b^	0.0001
**Weight of organs (g)**			
Liver	21.4 ± 1.04 ^b^	47.7 ± 2.27 ^a^	0.0001
Heart	8.4 ± 0.21 ^b^	10.1 ± 0.45 ^a^	0.0006
Gizzard	32.6 ± 1.36 ^b^	50.1 ± 0.45 ^a^	0.0001
Giblet	62.4 ± 2.02 ^b^	107.9 ± 2.94 ^a^	0.0001
Skin	113.0 ± 2.86	112.0 ± 4.17	0.8318
Testes	18.9 ± 1.12	Trace	
Liver yield (%)	1.2 ± 0.06 ^b^	2.7 ± 0.12 ^a^	0.0001
Heart yield (%)	0.5 ± 0.01 ^b^	0.6 ± 0.02 ^a^	0.0020
Gizzard yield (%)	1.9 ± 0.08 ^b^	2.9 ± 0.17 ^a^	0.0001
Giblet yield (%)	3.7 ± 0.19 ^b^	6.2 ± 0.20 ^a^	0.0001
Skin yield (%)	6.6 ± 0.15	6.4 ± 0.22	0.3578
Testes yield (%)	0.9 ± 0.11	Trace	
**Non-edible parts (g)**			
Head	72.9 ± 1.72 ^a^	47.4 ± 2.11 ^b^	0.0001
Shank/Feet	71.7 ± 1.31 ^a^	61.6 ± 3.88 ^b^	0.0061
Blood and feather	193.0 ± 5.87 ^a^	123.1 ± 9.77 ^b^	0.0001
Abdominal fat	Trace	20.0 ± 1.88	-
Head yield (%)	4.3 ± 0.09 ^a^	2.7 ± 0.07 ^b^	0.0001
Shank/Feet yield (%)	4.2 ± 0.07 ^a^	3.5 ± 0.18 ^b^	0.0001
Blood and feather yield (%)	11.3 ± 0.34 ^a^	7.0 ± 0.56 ^b^	0.0001
Abdominal fat yield (%)	-	1.1 ± 0.10	-

Data bearing different superscripts row wise differ significantly. NS-Non-significant.

**Table 3:** Meat quality parameters of Kadaknath and commercial broilers (Mean ± S.E.).

Traits	Genotypes		*p* Value
	Kadaknath	Broilers	
**pH of breast muscle**			
45 min	6.53 ± 0.02	6.53 ± 0.02	NS
24 h	6.47 ± 0.02 ^a^	6.38 ± 0.02 ^b^	0.0120
48 h	6.37 ± 0.02 ^a^	6.26 ± 0.02 ^b^	0.0001
Decline in pH after 24 h	0.06 ± 0.01 ^b^	0.15 ± 0.02 ^a^	0.0003
Decline in pH after 48 h	0.16 ± 0.02 ^b^	0.27 ± 0.02 ^a^	0.0002
**Meat weight (g)**			
Breast	174.50 ± 4.54 ^b^	305.90 ± 14.79 ^a^	0.0001
Thigh	147.30 ± 3.49	153.08 ± 6.26	0.3884
Drumstick	136.00 ± 3.20 ^a^	112.20 ± 4.25 ^b^	0.0001
Total of breast and legs	457.80 ± 7.59 ^b^	571.20 ± 24.10 ^a^	0.0001
**Bone weight (g)**			
Breast	71.60 ± 3.17	76.25 ± 3.73	0.3611
Thigh	35.95 ± 0.91 ^a^	31.92 ± 1.61 ^b^	0.0252
Drumstick	50.20 ± 1.40	50.17 ± 2.26	0.9895
Total of breast and legs	157.70 ± 4.18	158.30 ± 5.91	0.9347
**Meat–bone ratio**			
Breast	2.57 ± 0.17 ^b^	4.04 ± 0.13 ^a^	0.0001
Thigh	4.14 ± 0.14 ^b^	4.89 ± 0.23 ^a^	0.0002
Drumstick	2.74 ± 0.08 ^a^	2.26 ± 0.09 ^b^	0.0007
Total	2.94 ± 0.09 ^b^	3.61 ± 0.07 ^a^	0.0001

Data bearing different superscripts row wise differ significantly. NS; Non-significant.

**Table 4:** Meat colour characteristics of Kadaknath (Mean ± S.E.) after averaging 4 observations of each sample (Mean ± S.E.).

Traits	Genotypes		*p* Value
	Kadaknath	Broilers	
L* (lightness)			
Breast	42.44 ± 0.91 ^Ba^	50.92 ± 1.01 ^Aa^	0.0001
Thigh	35.37 ± 0.41 ^Bb^	47.48 ± 1.03 ^Ab^	0.0001
*p* value	0.0001	0.0266	
a* (redness)			
Breast	−0.61 ± 0.18 ^b^	−1.06 ± 0.19 ^b^	0.1081
Thigh	2.18 ± 0.29 ^a^	2.90 ± 0.45 ^a^	0.1924
*p* value	0.0001	0.0001	
b* (yellowness)			
Breast	6.23 ± 0.49 ^Ba^	8.99 ± 0.31 ^A^	0.0001
Thigh	4.17 ± 0.27 ^Bb^	8.98 ± 0.30 ^A^	0.0001
*p* value	0.0001	0.9699	
Hue (H*)			
Breast	−0.63 ± 0.29 ^b^	−1.18 ± 0.25 ^b^	0.1934
Thigh	0.95 ± 0.14 ^a^	1.26 ± 0.05 ^a^	0.1089
*p* value	0.0001	0.0001	
Chroma (C*)			
Breast	6.36 ± 0.45 ^Ba^	9.08 ± 0.30 ^A^	0.0001
Thigh	4.80 ± 0.33 ^Bb^	9.56 ± 0.28 ^A^	0.0001
*p* value	0.009	0.2637	

Data bearing different uppercase superscripts row wise and figures bearing different lowercase superscript column wise (under each trait) differ significantly.

**Table 5:** Proximate principles (on dry matter basis) of meat of Kadaknath and commercial broilers (Mean ± S.E.).

Traits (%)	Genotypes		*p* Value
	Kadaknath	Broilers	
Moisture	73.50 ± 0.14 ^b^	74.68 ± 0.22 ^a^	0.0001
Crude protein	24.24 ± 0.16 ^a^	23.09 ± 0.23 ^b^	0.0002
Lipids (Fat)	1.24 ± 0.09 ^b^	1.61 ± 0.06 ^a^	0.0075
Total ash	1.19 ± 0.03 ^b^	1.30 ± 0.04 ^a^	0.0310
Gross Energy * (kcal/kg)	4259 ± 16.4 ^b^	4420 ± 16.5 ^a^	0.0001

* Calculated, Data bearing different superscripts row wise differ significantly.

**Table 6:** Correlation coefficients among pH, colour attributes and proximate principles of breast meat (Kadaknath and broilers).

	pH45m	pH24h	pH48h	MBR	L*	a*	b*	Hue	C	M	P	F	A
**pH45m**	1												
**pH24h**	0.73 **	1											
**pH48h**	0.52 **	0.77 **	1										
**MBR**	−0.06	−0.21	−0.55 **	1									
L*	0.16	−0.21	−0.37 *	0.53 **	1								
a*	−0.22	−0.08	0.10	−0.22	−0.23	1							
b*	−0.05	−0.32	−0.42 *	0.32	0.77 **	0.17	1						
**Hue**	−0.17	−0.17	−0.05	−0.27	−0.33	0.68 **	0.03	1					
**C**	−0.04	−0.33	−0.43 *	0.32	0.78 **	0.13	0.99 **	0.03	1				
**M**	−0.01	−0.48 **	−0.61 **	0.34	0.51 **	−0.29	0.48 **	−0.10	0.51 **	1			
**P**	0.04	0.43 *	0.55 **	−0.35	−0.47 **	0.34	−0.47 **	0.21	−0.49 **	−0.89 **	1		
**F**	−0.16	−0.25	−0.26	0.47 **	0.24	−0.01	0.12	−0.12	0.11	0.06	−0.29	1	
**A**	−0.08	−0.20	−0.21	0.39 *	0.43 *	−0.15	0.23	−0.18	0.23	0.01	−0.04	0.19	1

* *p* ≤ 0.05, ** *p* ≤ 0.01. pH45m: pH at 45 min after slaughter, pH24h: pH at 24 h after slaughter, pH48h: pH at 48 h after slaughter, L*: lightness, b*: yellowness, a*: redness, MBR: meat–bone ratio, C: chroma, M: moisture, P: protein, F: fat, A: ash.

**Table 7:** Amino acid composition of Kadaknath and broiler meat on fresh weight basis (Mean ± S.E.).

Amino Acids (g/100 g of Meat)	Genotypes		*p* Value
	Kadaknath	Broilers	
**Indispensable amino acids**			
Arginine	2.00 ± 0.10	2.24 ± 0.11	0.1371
Histidine	0.030 ± 0.001 ^b^	0.036 ± 0.002 ^a^	0.0182
Leucine	1.14 ± 0.05 ^a^	0.93 ± 0.09 ^b^	0.0324
Lysine	4.00 ± 0.17	4.20 ± 0.22	0.4761
Methionine	2.52 ± 0.08 ^b^	3.03 ± 0.18 ^a^	0.0062
Phenylalanine	2.37 ± 0.07	2.52 ± 0.23	0.4498
Threonine	0.87 ± 0.04 ^b^	1.04 ± 0.08 ^a^	0.0422
Tryptophan	0.005 ± 0.0008 ^a^	0.002 ± 0.0003 ^b^	0.0222
Valine	2.48 ± 0.074	2.66 ± 0.15	0.2386
**Non-indispensable amino acids**			
Alanine	0.37 ± 0.04 ^a^	0.12 ± 0.01 ^b^	0.0002
Aspargine	0.04 ± 0.003 ^a^	0.02 ± 0.002 ^b^	0.0001
Aspartic acid	0.10 ± 0.013 ^a^	0.03 ± 0.003 ^b^	0.0002
Citrulline	0.02 ± 0.002 ^a^	0.006 ± 0.0009 ^b^	0.0001
Cysteine	0.024 ± 0.004 ^a^	0.007 ± 0.0008 ^b^	0.0038
Glutamic acid	2.78 ± 0.12 ^a^	1.89 ± 0.14 ^b^	0.0001
Glycine	0.002 ± 0.0002 ^a^	0.0006 ± 0.000005 ^b^	0.0002
Proline	2.25 ± 0.11 ^a^	1.71 ± 0.12 ^b^	0.0035
Serine	0.69 ± 0.05 ^a^	0.31 ± 0.04 ^b^	0.0001
Tyrosine	1.15 ± 0.07	1.35 ± 0.15	0.1981
Tasty amino acids *	4.74 ± 0.21 ^a^	3.38 ± 0.24 ^b^	0.0003

* Tasty amino acids: sum of alanine, asparagine, glutamic acid, glycine, serine and threonine, data bearing different superscripts row wise differ significantly.

## Data Availability

The data of the experiment is available from the authors, and it is deposited at ICAR-Krishi data repository. Available online: http://krishi.icar.gov.in/jspui/handle/123456789/73761.

## Supplementary Materials

The following supporting information can be downloaded at: https://www.mdpi.com/article/10.3390/foods11223603/s1, Figure S1: Thigh muscles of Kadaknath (left) and broilers (right), (a) Out-side view (b) Inside view.
