# Supplementary figures and images for: Carcass Characteristics, Meat Quality and Nutritional Composition of Kadaknath, a Native Chicken Breed of India
Source: Foods. 2022 Nov 11;11(22):3603. doi: 10.3390/foods11223603 (PMC9689593; doi:10.3390/foods11223603)

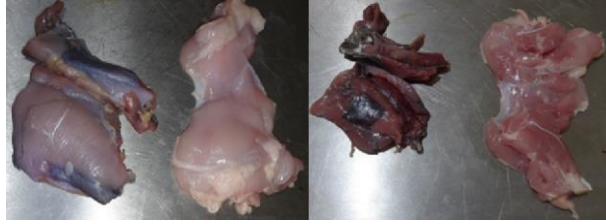

**Figure S1.** Thigh muscles of Kadaknath (left) and broilers (right), (a) Out-side view (b) Inside view.

Supplement: Supplementary file 1 [file foods-11-03603-s001.zip › foods-1938855-supplementary.pdf]
